# Supplementary material for: Connecting chemical structure to single cell signaling profiles
Source: Commun Biol. 2025 Jul 31;8:1137. doi: 10.1038/s42003-025-08545-3 (PMC12313981; doi:10.1038/s42003-025-08545-3)
Supplement: Supplementary file 1 — Supplemental Information [file 42003_2025_8545_MOESM1_ESM.pdf]

Thirman et. al. – Supplementary Figure 1

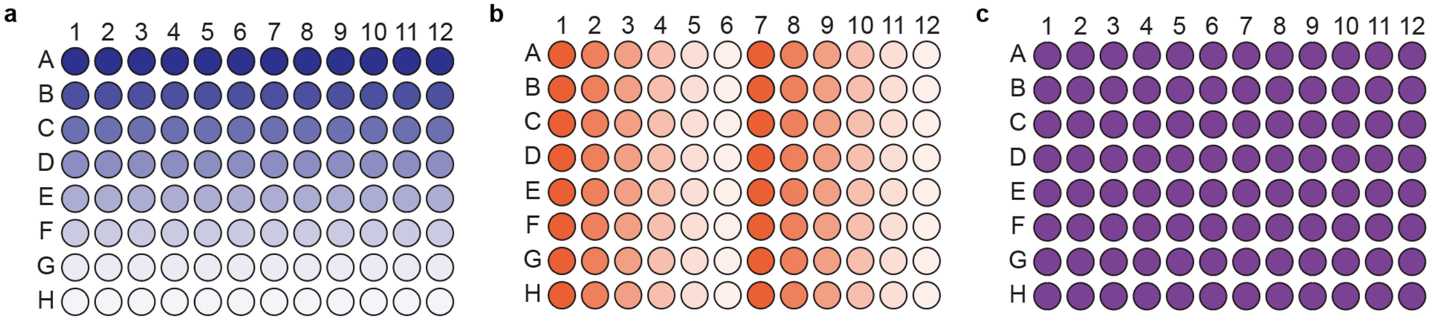

Supplementary Figure 1 – Fluorescent cell barcoding uses a combination of varying concentrations of Pacific Orange, Pacific Blue, and Alexa Fluor 750 to assign each well a unique combination of dyes. a) Rows of cells were fluorescently barcoded using 8 different concentrations of Pacific Blue. **b)** Columns of cells were fluorescently barcoded using 2 sets of 6 different concentrations of Pacific Orange. **c)** Alexa Fluor 750 was used at a single concentration as a dye uptake control. These three dyes are all used together on one plate to provide each well with a unique signature of Pacific Orange, Pacific Blue, and Alexa Fluor 750.

Supplementary Table 1 – Rocaglate Set evaluated based on example metrics of bioactivity, specific marker activity, selective activity, signature profile, and proximal target engagement

Compound	Subclass	Bioactivity, Ex.: $\sum_{All\ markers} \lvert Fold\ change\ MFI/\ Veh \rvert$	γH2AX Activity, Ex.: $\%\gamma H2AX+$	Selective Activity, Ex.: $log_2(\frac{\%\gamma H2AX+MV411}{\%\gamma H2AX+PBMC})$	Signature Profile, Ex.: $\frac{95^{th}\ Percentile\ \gamma H2AX}{95^{th}\ Percentile\ p\text{-}4EBP1}$	Proximal Target Engagement , Ex. : $eIF4A1\ Clamping*$
CMLD010510	1	6.96	6.17	-0.91	0.438	68.1
CMLD011880	1	7.43	8.42	-1.22	0.625	22.4
CMLD010508	1	5.13	9.01	-1.30	0.438	79.8
CMLD010583	1	2.1	12.1	0.99	0.359	13.3
CMLD010584	1	2.0	14.5	1.66	0.360	21.5
CMLD013586	1	2.0	15.4	1.39	0.467	71.3
CMLD011872	1	2.5	16.8	1.31	0.442	11.2
CMLD010536	1	2.8	20.2	1.01	0.559	38.7
BUCMD00557	1	2.9	20.3	1.64	0.460	
CMLD013210	1	2.4	20.6	1.55	0.522	70.7
CMLD013585	1	4.9	21.6	1.32	0.640	85.9
CMLD010513	1	2.9	21.8	0.87	0.762	71.5
CMLD011352	1	2.6	26.6	1.59	0.446	60.1
CMLD012655	1	2.3	27.3	1.52	0.498	
CMLD010853	1	2.0	28.4	2.12	0.449	70.9
CMLD010511	1	3.0	29.8	1.65	0.777	38.5
CMLD010515	1	2.4	31.2	1.42	0.606	94.6
CMLD012410	1	3.2	34.3	1.39	0.439	61.3
CMLD011891	1	2.2	35.5	2.38	0.515	63.7
CMLD011890	1	2.2	39.5	2.23	0.544	85.7
BUCMD00513	2	7.8	3.0	-2.82	2.01	71.9
CMLD012824	2	6.7	5.3	-2.14	0.534	116.8
CMLD013608	2	4.9	5.9	-1.92	0.337	72.1
CMLD013334	2	5.0	6.0	-1.81	0.371	79.1
CMLD012611	2	5.6	6.1	-1.86	0.373	95.4
CMLD013333	2	4.6	8.7	-1.32	0.361	56.4
CMLD012565	2	4.4	19.4	-0.04	0.440	65.6
CMLD012600	2	3.0	36.4	0.85	0.514	70.1
CMLD013626	3	1.8	20.7	1.71	0.346	51.6
CMLD011866	3	2.4	21.7	1.61	0.506	70.3
CMLD011839	3	1.9	27.0	2.20	0.454	51
CMLD013347	3	2.7	28.9	1.90	0.595	16.1
CMLD012390	3	2.0	31.2	2.40	1.27	66.5
CMLD013348	3	2.0	32.1	2.75	1.00	58.3
CMLD010482	3	2.4	33.5	1.91	0.787	
CMLD010582	3	2.8	36.1	1.98	0.627	-0.7
CMLD013342	3	2.1	38.5	2.61	1.14	48.3

The Rocaglate Set organized according to rocaglate subclass and evaluated for activity using the following flagship examples for each metric: bioactivity as quantified by the sum across all markers of the absolute value of median fluorescence intensity over vehicle (see **Figure 3a**), specific marker activity as quantified by $\%\gamma H2AX+$ in MV411, selective activity as quantified by $log_2(\%\gamma H2AX+MV411/\%\gamma H2AX+PBMC)$, signature profile quantified by $95^{th}\ Percentile\ \gamma H2AX/95^{th}\ Percentile\ p\text{-}4EBP1$ in MV411, and proximal target engagement by eIF4A1 clamping values. All metrics are all colored on a scale from dark blue, indicating the lowest value, grey indicating the midpoint value, and yellow indicating the highest value per column.
*eIF4A1 clamping values are from Naineni et al., (PMCID: PMC10187672) [1]. Potency values that were absent from published data have a dash.

Supplementary Table 2 – Flow cytometry reagents

Reagent	Detect	Clone	Fluorochrome	Vendor	Cat. #	Dilution	Readout	Div. Set	Roc. Set	Dose Response	SAR
Antibody	c-CAS3	C92-605	PE	BD Biosciences	570185	1:10	Apoptosis [2]	X			
Antibody	c-CAS3	C92-605.rMAb	BV650	BD Biosciences	570189	1:200	Apoptosis [2]				X
Antibody	Ki67	B56	BV786	BD Biosciences	563756	1:40	Cell proliferation [3,4]		X	X	X
Antibody	p-S6 S235/236	D57.2.2E	Ax594	Cell Signaling Technology	9865	1:200	mTOR+MAPK/ ERK activation [5 6]		X	X	
Antibody	p-S6 S235/236	D57.2.2E	Ax647	Cell Signaling Technology	4851	1:400	mTOR+MAPK/ ERK activation [5 6]	X			
Antibody	p-S6 S240/244	D68F8	Ax488	Cell Signaling Technology	5018	1:4000	mTOR activation [5 6]		X	X	X
Antibody	p-AKT S473	D9E	APC	Cell Signaling Technology	11962	1:100	Upstream of mTOR [7,8]		X		
Antibody	p-LCK Y505	SRRCHA	PerCP-eFluor 710	ThermoFisher	46-9076-42	1:40	Src-family kinase [9]		X		
Antibody	γH2AX S139	N1-431	PerCP-Cy5.5	BD Biosciences	564718	1:40	DNA damage [10]	X	X	X	X
Antibody	p-STAT3 S727	49/p-Stat3	PE	BD Biosciences	558557	1:30	Transcriptional Activity [11]		X	X	
Antibody	p-STAT5 Y694	SRBCZX	PE-eFluor610	Invitrogen	61-9010-42	1:40	Cell survival [12 13]		X	X	
Antibody	p-ERK T202/ Y204	6B8B69	PE-Cy5	Biolegend	369514	1:40	MAPK activation [14,15]		X	X	
Antibody	p-HH3 S28	HTA28	PE-Cy7	Biolegend	641011	1:1000	M-phase cell cycle [16]	X	X	X	
Antibody	p-HH3 S10	D2C8	PE	Cell Signaling Technology	5764S	1:200	M-phase cell cycle [16]				X
Antibody	p-4EBP1 T37/46	236B4	Ax647	Cell Signaling Technology	5123S	1:200	Translation [17]		X	X	X
NHS dye	Primary amines		Pacific Blue	ThermoFisher Scientific	P10163	Described in Methods		X	X	X	X
NHS dye	Primary amines		Pacific Orange	ThermoFisher Scientific	P30253	Described in Methods		X	X	X	X
NHS dye	Primary amines		Ax750	ThermoFisher Scientific	A20011	Described in Methods		X	X	X	X
NHS dye	Primary amines		Ax700	ThermoFisher Scientific	A20010	Described in Methods		X	X	X	X

Table lists functional protein states used for measuring specific pathways by flow cytometry, their clone, fluorochrome, vendor source, dilution used in assay, their role in various intracellular signaling pathways, and the corresponding panel where they were used. Modeled after Balsamo et. al. [18].

Supplementary Table 3 – Positive and negative control compounds

Compound	Conc.	Purpose
Dimethyl sulfoxide (DMSO)		Negative control
staurosporine	1 μM	c-CAS3 + γH2AX pos. control
etoposide	10 μM	γH2AX + c-CAS3 pos. control
**CMLD010335**	10 μM	Rocaglate control
rapamycin	0.01 μM	p-S6 suppression pos. control
Nocodazole	4 μM	G2 cell cycle arrest + p-HH3 pos. control
aphidicolin	4 μM	G1 cell cycle arrest + p-HH3 pos. control

Table lists compounds used as positive and negative controls for specific pathway measurements made by flow cytometry, vendor source, final concentration, and a short description of their purpose.

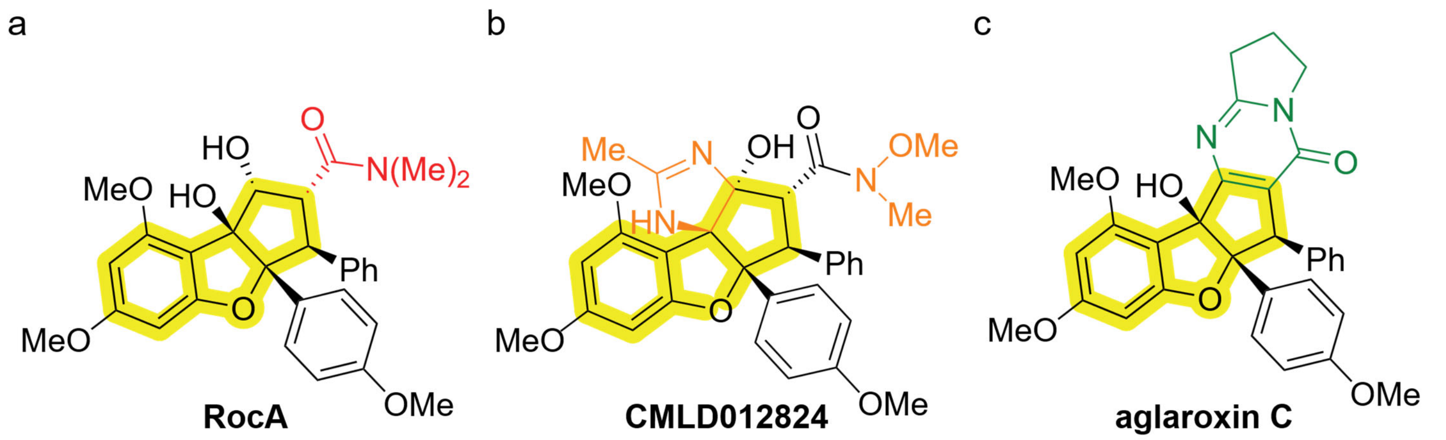

Supplementary Figure 2 – RocA, CMLD012824, and aglaroxin C were exemplar rocaglates for their respective subclasses. a) Chemical structure for rocaglamide (RocA), a regular rocaglate (RR). R group is colored red based on classification as RR. **b)** Chemical structure for CMLD012824, an amidino rocaglate (ADR). Ring fusion is colored orange based on classification as ADR. **c)** Chemical structure for aglaroxin C, a rocaglate pyrimidinone. Ring fusion is colored in green based on classification as RP.

Thirman et. al. – Supplementary Figure 3

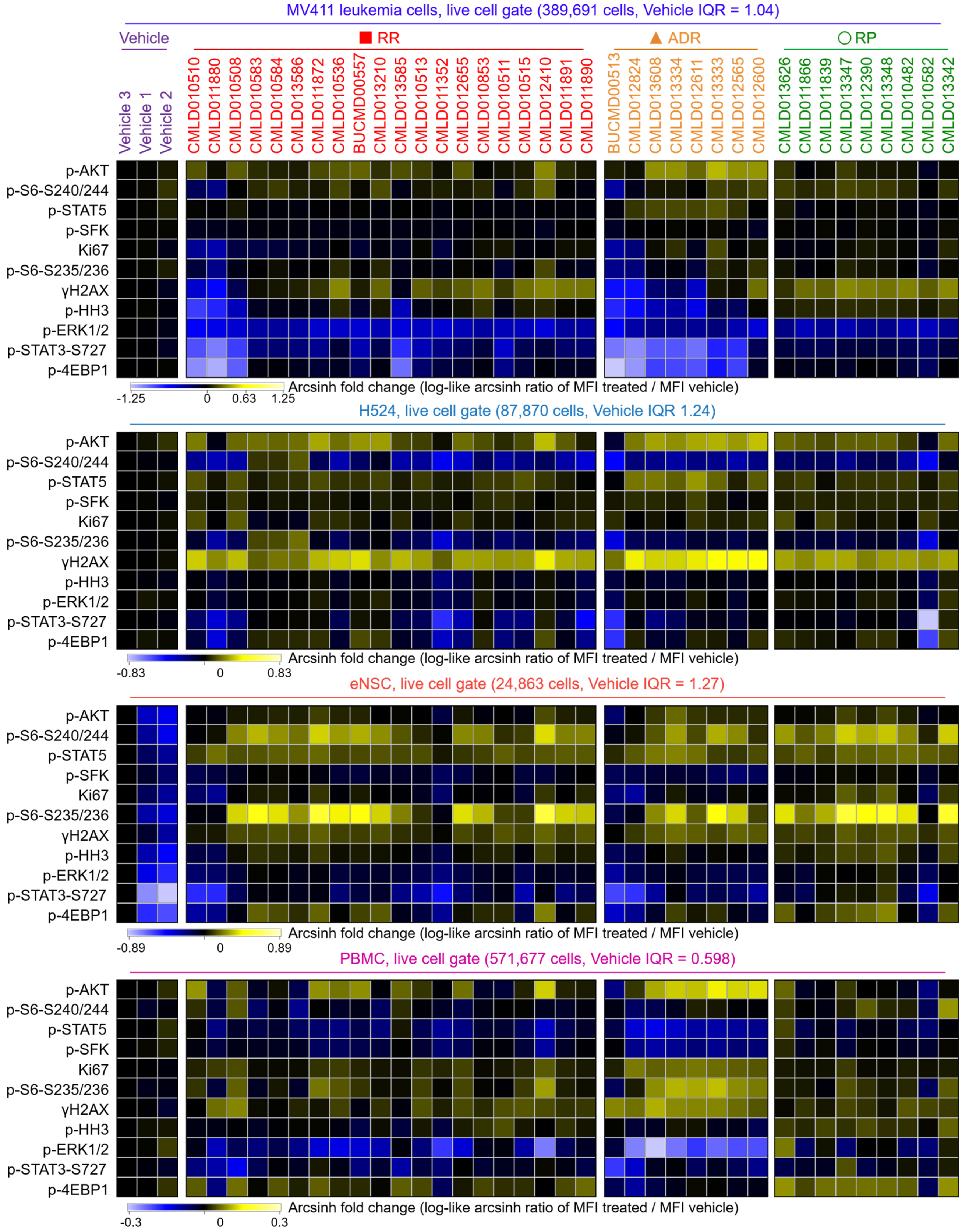

Supplementary Figure 3 – Rocaglates demonstrate contrasting pharmacodynamics across subclass in MV411 and PBMC in comparison with H524 and eNSC. Heatmaps depicting the arcsinh ratio of the median fluorescence intensity (MFI) for each compound (listed on top) and readout (listed on left) by the MFI of Vehicle 3 for MV411, H524, eNSC, and PBMC, respectively. Cells on the heatmap range from light blue for the lowest values to bright yellow for the highest values. Compounds are grouped and colored according to the rocaglate subclass listed on the top of the plot.

Thirman et. al. – Supplementary Figure 4

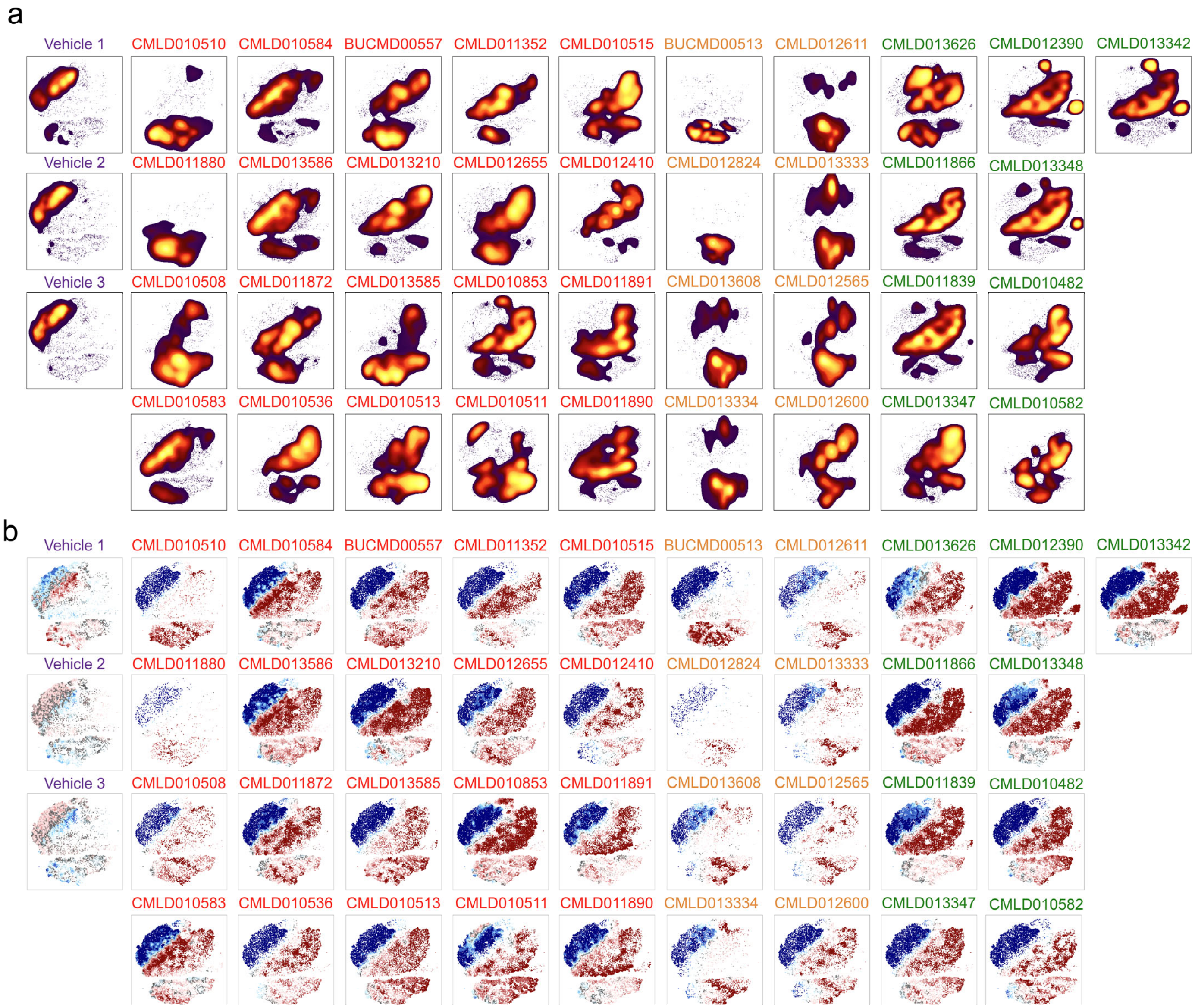

Supplementary Figure 4 - Rocaglates had distinct signature profiles in leukemia. a) t-SNE plots depicting the result of performing a t-SNE analysis on the entire pre-processed MV411 dataset and dividing based on compound. **b)** T-REX plots depict regions of significant difference between the t-SNE of one compound vs. the pooled set of vehicle-treated cells in MV411. For both **a)** and **b)** compound names are colored according to rocaglate structural subclass.

Thirman et. al. – Supplementary Figure 5

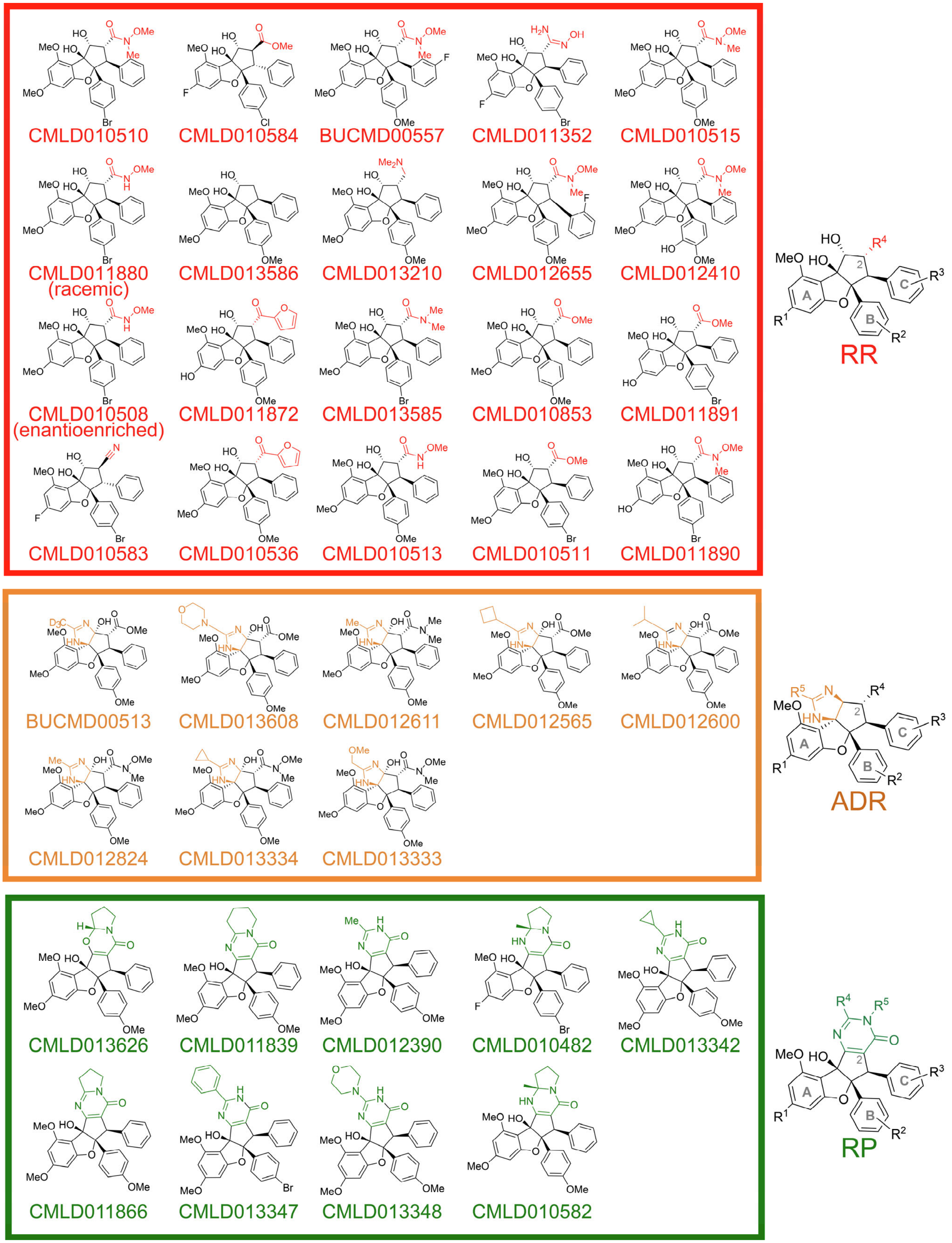

Supplementary Figure 5 - The 37 rocaglates were organized into three structural subclasses. The structures of the 37 rocaglates are organized into boxes according to membership to one of three subclasses. Boxes, compound names, and R groups are colored according to rocaglate subclass. The defining structural scheme for each subclass is shown to the right of each box.

Thirman et. al. – Supplementary Figure 6

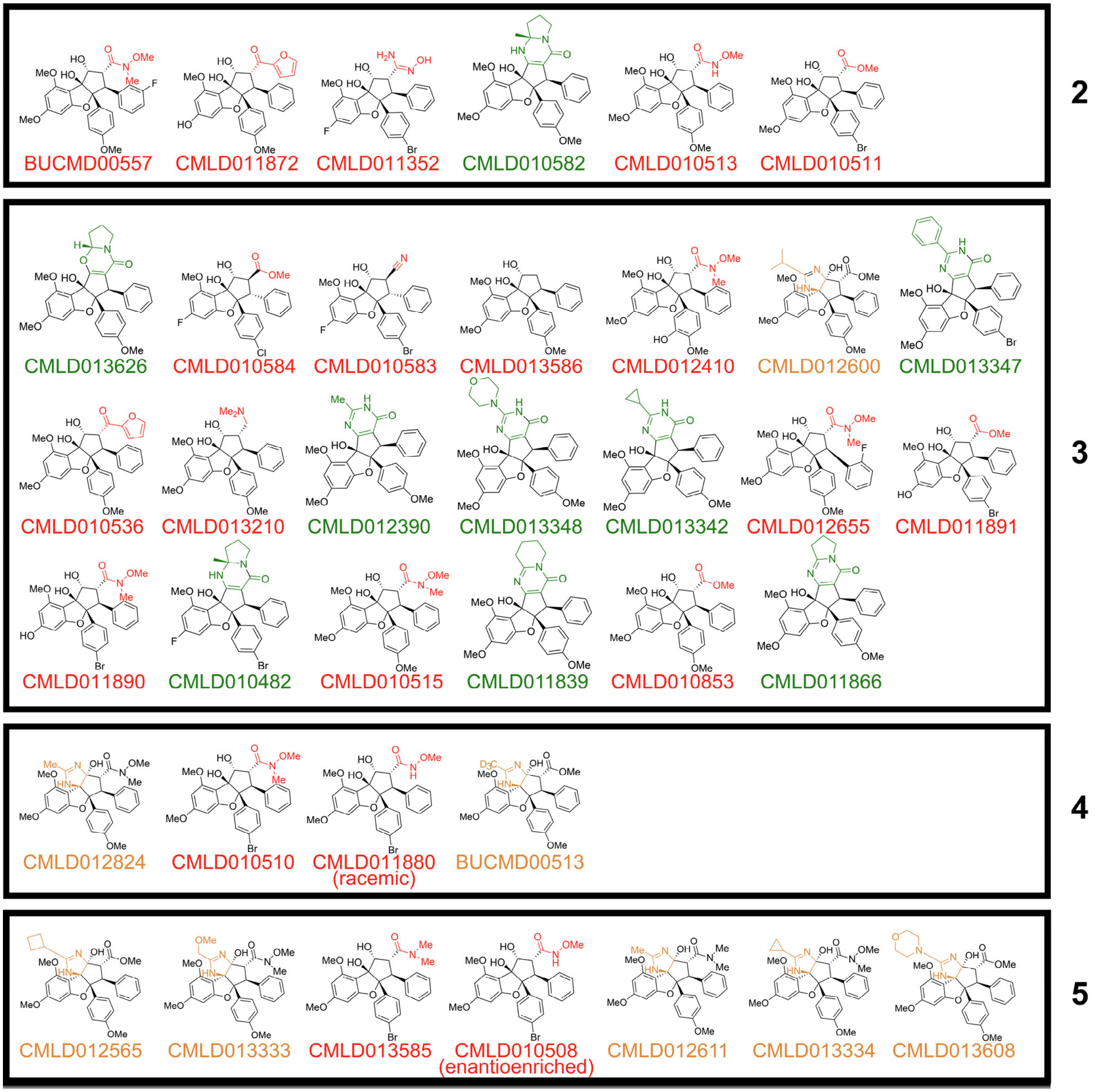

Supplementary Figure 6 – Dendrogram clusters provided deeper insight into potential machine-learned structure-activity relationships. The structures of the 37 rocaglates are organized according to the dendrogram clusters formed based on transformed median fluorescence intensity in **Figure 3c**. Compound names and R groups are colored according to rocaglate structural subclass. The cluster number from **Figure 3c** is listed on the right side of each box.

Thirman et. al. – Supplementary Figure 7

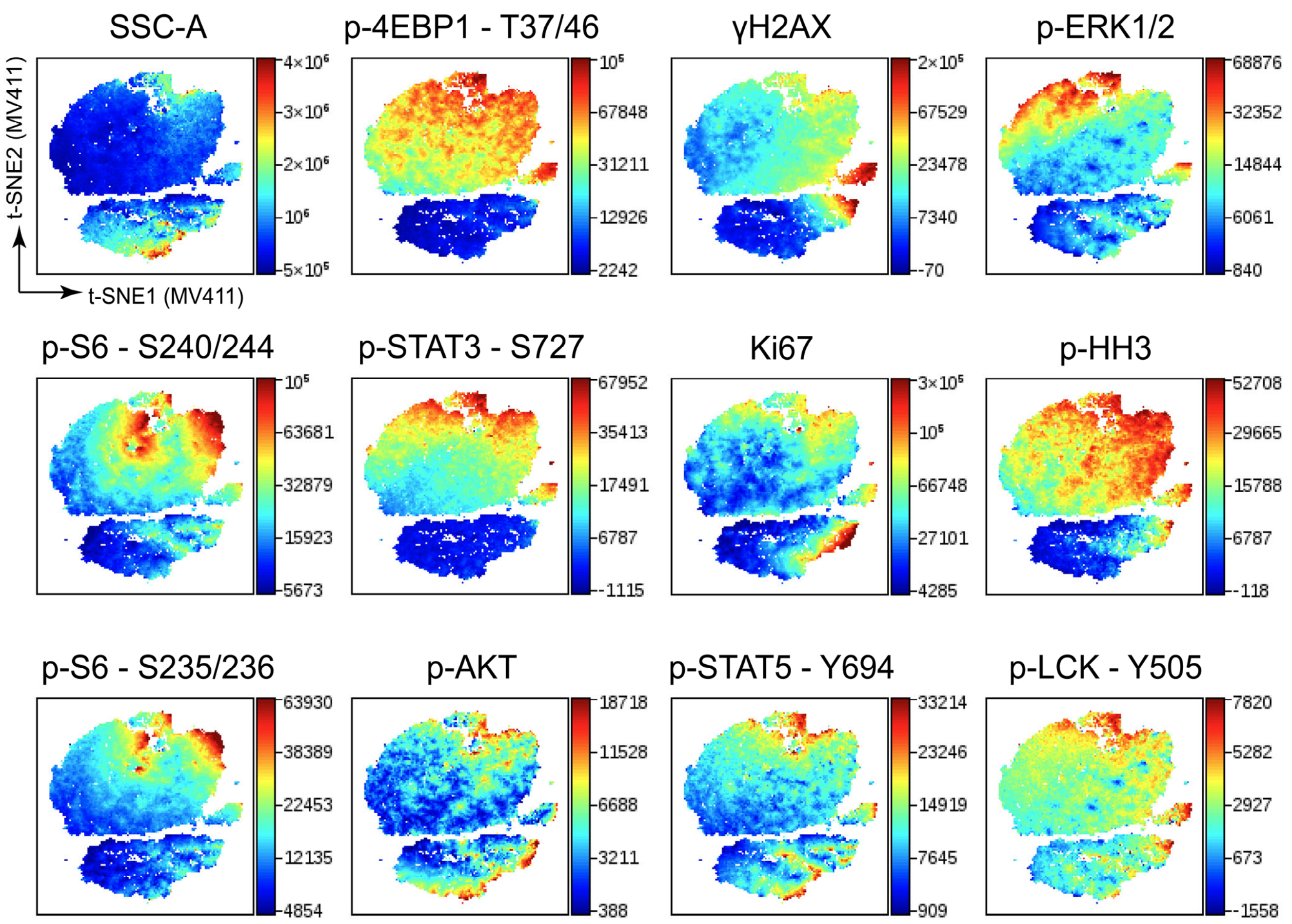

Supplementary Figure 7 - Heterogeneity existed at the single-cell level across functional readouts. Plot depicting the result of performing a t-SNE analysis on the entire pre-processed Rocaglate Set MV411 data and coloring based on protein measurements for each of the 11 functional readouts tested (and SSC-A). Each readout is on its own scale as seen in the legend on the right side of each plot.

Thirman et. al. – Supplementary Figure 8

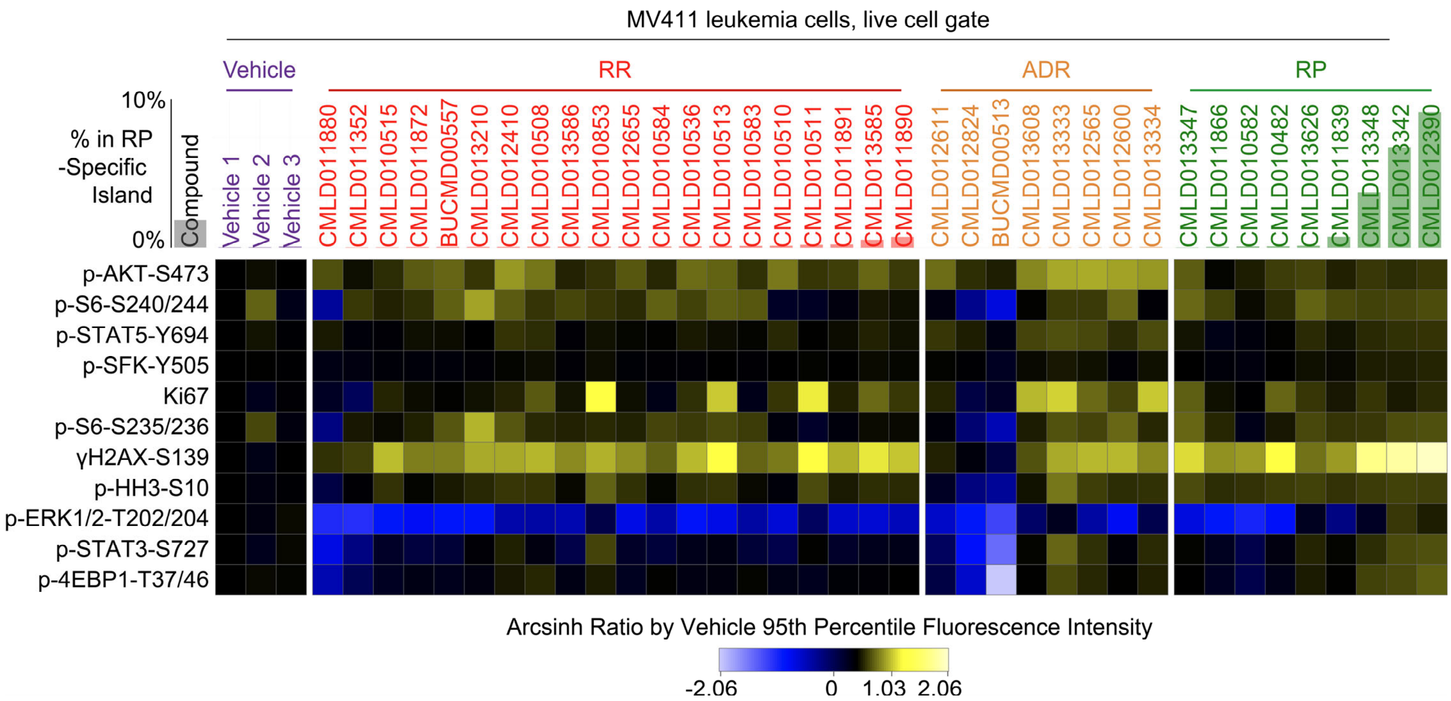

Supplementary Figure 8 – The RP Island was associated with high γH2AX and p-4EBP1 and low p-ERK. Heatmap depicting the arcsinh ratio of the 95th percentile fluorescence intensity for each compound (listed on top of heatmap) and readout (listed left of heatmap) by the median fluorescence intensity of Vehicle 1. Cells on the heatmap range from light blue for the lowest values to bright yellow for the highest values. Compounds are grouped and colored according to the rocaglate subclass listed on the top of the plot. A bar plot depicting the percentage of cells in the RP island for each compound is shown behind each respective compound name.

Thirman et. al. – Supplementary Figure 9

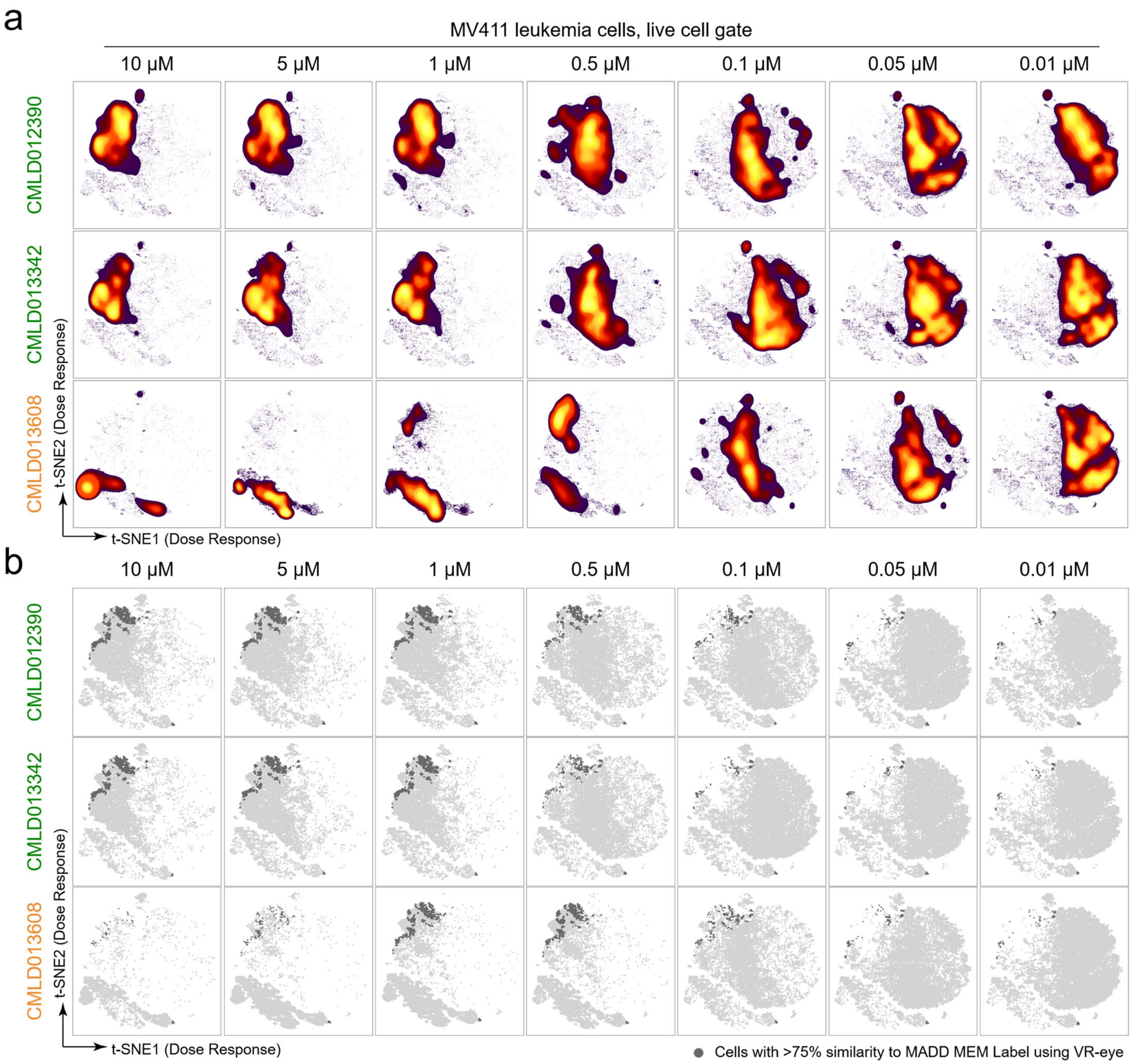

Supplementary Figure 9 - MADD signaling profile was induced by two of the exceptional RPs in a dose dependent manner. a) t-SNE plot from dose response experiment performed on MV411 leukemia cells for two RPs (**CMLD012390** and **CMLD013342**) and one ADR (**CMLD013608**) divided based on compound and dose. **b)** Cells with >75% similarity to the MADD signaling profile MEM label from **Figure 5c** overlaid on t-SNE plots from **Supplementary Figure 9a**.

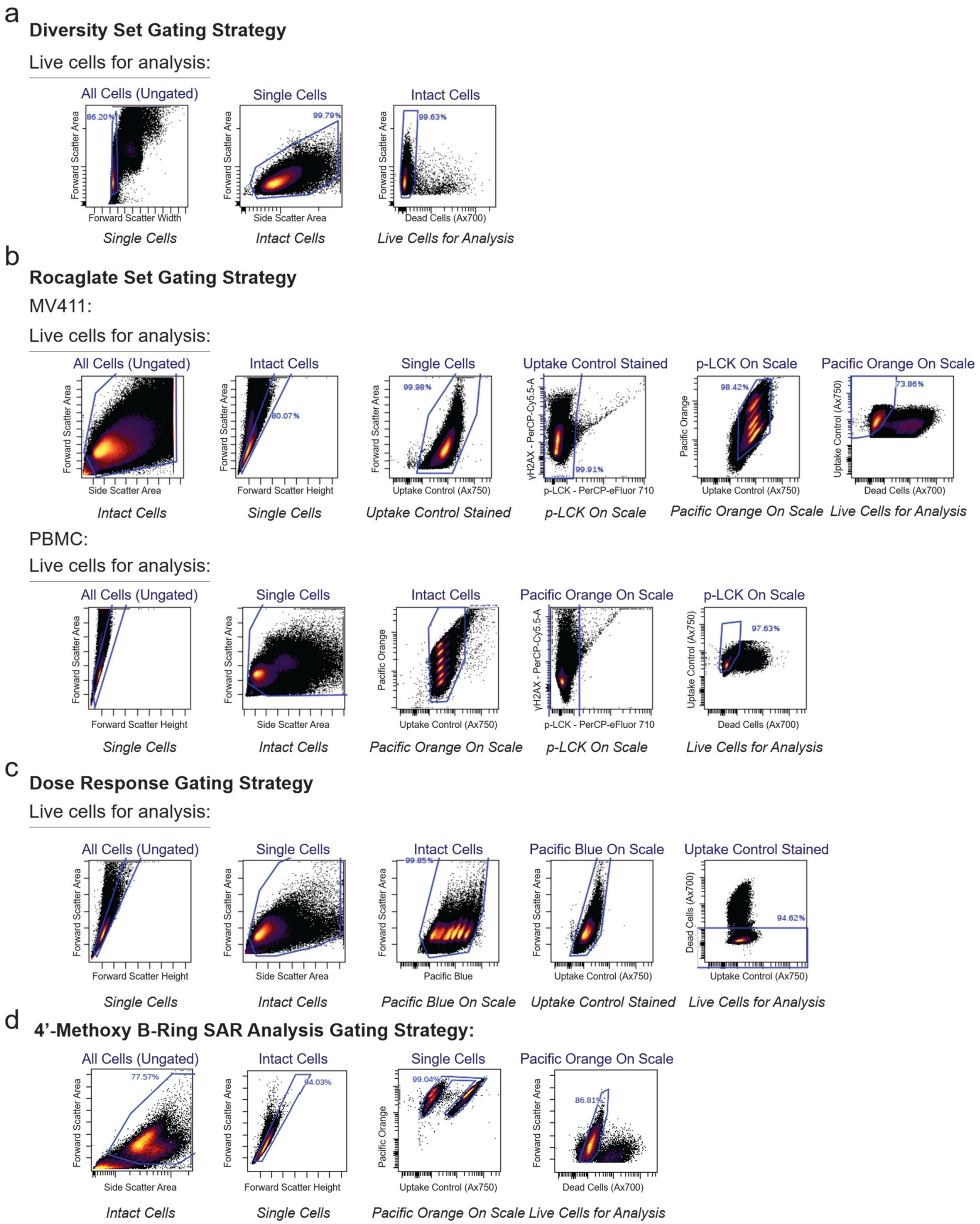

Supplementary Figure 10 - Gating was used to identify intact, single, live cells for analysis. a) **<u>Diversity Set</u>** gating strategy demonstrated on flow cytometry density dot plots. Columns 1-6 of Plate A are used as reference cells. **b)** **<u>Rocaglate Set</u>** gating strategy demonstrated on flow cytometry density dot plots for MV411 and PBMC. **c)** Gating strategy for dose response data demonstrated on flow cytometry density dot plots. **CMLD012390** data are used as reference cells. **d)** Gating strategy for 4'-methoxy B-ring SAR analysis demonstrated on flow cytometry density dot plots.

References

1 Naineni, S. K. *et al.* Exploring the targeting spectrum of rocaglates among eIF4A homologs. *RNA (Cambridge)* **29**, 826-835 (2023). https://doi.org:10.1261/rna.079318.122
2 Crowley, L. C. & Waterhouse, N. J. Detecting Cleaved Caspase-3 in Apoptotic Cells by Flow Cytometry. *Cold Spring Harb Protoc* **2016** (2016). https://doi.org:10.1101/pdb.prot087312
3 Uxa, S. *et al.* Ki-67 gene expression. *Cell Death & Differentiation* **28**, 3357-3370 (2021). https://doi.org:10.1038/s41418-021-00823-x
4 Miller, I. *et al.* Ki67 is a Graded Rather than a Binary Marker of Proliferation versus Quiescence. *Cell Rep* **24**, 1105-1112.e1105 (2018). https://doi.org:10.1016/j.celrep.2018.06.110
5 Magnuson, B., Ekim, B. & Fingar, D. C. Regulation and function of ribosomal protein S6 kinase (S6K) within mTOR signalling networks. *Biochem J* **441**, 1-21 (2012). https://doi.org:10.1042/bj20110892
6 Roux, P. P. *et al.* RAS/ERK Signaling Promotes Site-specific Ribosomal Protein S6 Phosphorylation via RSK and Stimulates Cap-dependent Translation *. *Journal of Biological Chemistry* **282**, 14056-14064 (2007). https://doi.org:10.1074/jbc.M700906200
7 Blix, E. S. *et al.* Phospho-specific flow cytometry identifies aberrant signaling in indolent B-cell lymphoma. *BMC Cancer* **12**, 478 (2012). https://doi.org:10.1186/1471-2407-12-478
8 Irish, J. M. *et al.* B-cell signaling networks reveal a negative prognostic human lymphoma cell subset that emerges during tumor progression. *Proc Natl Acad Sci U S A* **107**, 12747-12754 (2010). https://doi.org:10.1073/pnas.1002057107
9 Takata, M. *et al.* Tyrosine kinases Lyn and Syk regulate B cell receptor-coupled Ca2+ mobilization through distinct pathways. *The EMBO Journal* **13**, 1341-1349-1349 (1994). https://doi.org:https://doi.org/10.1002/j.1460-2075.1994.tb06387.x
10 Mah, L. J., El-Osta, A. & Karagiannis, T. C. γH2AX: a sensitive molecular marker of DNA damage and repair. *Leukemia* **24**, 679-686 (2010). https://doi.org:10.1038/leu.2010.6
11 Igelmann, S., Neubauer, H. A. & Ferbeyre, G. STAT3 and STAT5 Activation in Solid Cancers. *Cancers (Basel)* **11** (2019). https://doi.org:10.3390/cancers11101428
12 Wingelhofer, B. *et al.* Pharmacologic inhibition of STAT5 in acute myeloid leukemia. *Leukemia* **32**, 1135-1146 (2018). https://doi.org:10.1038/s41375-017-0005-9
13 Kotecha, N. *et al.* Single-cell profiling identifies aberrant STAT5 activation in myeloid malignancies with specific clinical and biologic correlates. *Cancer Cell* **14**, 335-343 (2008). https://doi.org:10.1016/j.ccr.2008.08.014
14 Roskoski, R., Jr. ERK1/2 MAP kinases: structure, function, and regulation. *Pharmacol Res* **66**, 105-143 (2012). https://doi.org:10.1016/j.phrs.2012.04.005
15 Mendoza, M. C., Er, E. E. & Blenis, J. The Ras-ERK and PI3K-mTOR pathways: cross-talk and compensation. *Trends Biochem Sci* **36**, 320-328 (2011). https://doi.org:10.1016/j.tibs.2011.03.006
16 Hans, F. & Dimitrov, S. Histone H3 phosphorylation and cell division. *Oncogene* **20**, 3021-3027 (2001). https://doi.org:10.1038/sj.onc.1204326
17 Gingras, A. C. *et al.* Regulation of 4E-BP1 phosphorylation: a novel two-step mechanism. *Genes Dev* **13**, 1422-1437 (1999). https://doi.org:10.1101/gad.13.11.1422
18 Balsamo, J. A. *et al.* An immunogenic cell injury module for the single-cell multiplexed activity metabolomics platform to identify promising anti-cancer natural products. *J Biol Chem* **298**, 102300 (2022). https://doi.org:10.1016/j.jbc.2022.102300
